# Combination of Intra-Articular and Intraosseous Injections of Platelet Rich Plasma for Severe Knee Osteoarthritis: A Pilot Study

**DOI:** 10.1155/2016/4868613

**Published:** 2016-07-04

**Authors:** Mikel Sánchez, Diego Delgado, Pello Sánchez, Emma Muiños-López, Bruno Paiva, Froilán Granero-Moltó, Felipe Prósper, Orlando Pompei, Juan Carlos Pérez, Juan Azofra, Sabino Padilla, Nicolás Fiz

**Affiliations:** ^1^Arthroscopic Surgery Unit, Hospital Vithas San Jose, C/Beato Tomás de Zumarraga 10, 01008 Vitoria-Gasteiz, Spain; ^2^Arthroscopic Surgery Unit Research, Hospital Vithas San Jose, C/Beato Tomás de Zumarraga 10, 01008 Vitoria-Gasteiz, Spain; ^3^Cell Therapy Area, Clínica Universidad de Navarra, Avenida de Pío XII 36, 31008 Pamplona, Spain; ^4^Center for Applied Medical Research, Avenida de Pío XII 55, 31008 Pamplona, Spain; ^5^Orthopaedic Surgery and Traumatology Department, Clínica Universidad de Navarra, Avenida de Pío XII 36, 31008 Pamplona, Spain; ^6^Hematology Department, Clínica Universidad de Navarra, Avenida de Pío XII 36, 31008 Pamplona, Spain; ^7^Fundacion Eduardo Anitua, C/Jose María Cagigal 19, 01007 Vitoria-Gasteiz, Spain

## Abstract

The aim of this study was to assess a novel approach to treating severe knee osteoarthritis by targeting synovial membrane, superficial articular cartilage, synovial fluid, and subchondral bone by combining intra-articular injections and intraosseous infiltrations of platelet rich plasma. We explored a new strategy consisting of intraosseous infiltrations of platelet rich plasma into the subchondral bone in combination with the conventional intra-articular injection in order to tackle several knee joint tissues simultaneously. We assessed the clinical outcomes through osteoarthritis outcome score (KOOS) and the inflammatory response by quantifying mesenchymal stem cells in synovial fluid. There was a significant pain reduction in the KOOS from baseline (61.55 ± 14.11) to week 24 (74.60 ± 19.19), after treatment (*p* = 0.008), in the secondary outcomes (symptoms, *p* = 0.004; ADL, *p* = 0.022; sport/rec., *p* = 0.017; QOL, *p* = 0.012), as well as VAS score (*p* < 0.001) and Lequesne Index (*p* = 0.008). The presence of mesenchymal stem cells in synovial fluid and colony-forming cells one week after treatment decreased substantially from 7.98 ± 8.21 MSC/*μ*L to 4.04 ± 5.36 MSC/*μ*L (*p* = 0.019) and from 601.75 ± 312.30 to 139.19 ± 123.61  (*p* = 0.012), respectively. Intra-articular injections combined with intraosseous infiltrations of platelet rich plasma reduce pain and mesenchymal stem cells in synovial fluid, besides significantly improving knee joint function in patients with severe knee osteoarthritis. This trial is registered on EudraCT with the number 2013-003982-32.

## 1. Introduction

Knee osteoarthritis (KOA) is a mechanically induced, cytokine and enzyme-mediated disorder comprising different phases and phenotypes, with pain as the clinical hallmark of the disease [1]. This diarthrodial joint is a complex biological system where articular cartilage (AC), an aneural and avascular tissue, lies functionally sandwiched between two highly vascularized and innervated tissues, namely, synovial membrane (SM), which produces synovial fluid (SF), and subchondral bone (SB), both endowed with heat receptors, chemoreceptors, and mechanoreceptors. Nociceptive stimuli, coming from a microenvironment undergoing nonphysiological mechanical loading and/or proinflammatory cytokines and damage-associated molecular patterns (DAMPS), might initially lead to peripheral and eventually both peripheral and neuropathic pain traits by mechanisms yet to be fully identified [2–4]. Moreover, the aggression to these tissues causes a surge of mesenchymal stem cells (MSCs) in SF as a part of tissue response to injury [5, 6].

In patients with severe OA, the subchondral bone undergoes changes which include microcracks and structural defects, vascularization of channels, nerve growth, and a progressive replacement of the subchondral marrow with fibroneurovascular mesenchymal tissue changes which underpin the increasingly recognized crosstalk and pathway for direct transport of growth factors such as transforming growth factor B (TGF*β*) and nerve growth factor (NGF) and even for cells such as macrophages and MSCs between the subchondral bone and articular cartilage [7–10].

As it is yet to be established which of the joint tissues or structures is the primary driver of KOA and therapeutic strategies that solely target one cell or tissue may well prove to fail, it is advisable that approaches to treating KOA should aim at reaching several joint tissues [11].

In patients with severe KOA, platelet rich plasma (PRP) and many bioactive mediators present in it have been shown to exert positive effects on the homeostasis of joint tissues through chondroprotective, anabolic, anti-inflammatory, and immunomodulatory effects and to substantially reduce pain, relieve joint stiffness, and improve physical function [12–20]. The aim of this study is to assess a novel approach to treating severe KOA, targeting synovial membrane, superficial articular cartilage, synovial fluid, and subchondral bone by combining intra-articular injections and intraosseous infiltrations of PRP. The hypothesis was that the addition of intraosseous injections of PRP directly into the subchondral bone to conventional intra-articular treatment would achieve a positive effect on patients with severe KOA.

## 2. Patients and Methods

The study was carried out in accordance with the international standard on clinical trials: Real Decreto 223/2004, Declaration of Helsinki in its latest revised version (Fortaleza, Brazil; 2013), and Good Clinical Practice Regulations (International Conference for Harmonization). The study protocol was reviewed and approved by the Reference Ethics Committee. All patients provided written informed consent before entry into the study.

### 2.1. Patient Selection

Nineteen patients were initially assessed for eligibility. Patients were considered eligible if they were aged between 40 and 77 years and presented severe knee osteoarthritis according to radiographic confirmation (Ahlbäck degrees 3 and 4, on a scale from 1 to 4, with the highest degrees indicating more severe OA). Finally, 14 patients were enrolled in the study from January 2014. The inclusion and exclusion criteria that patients had to meet in order to be included in this study are as follows.

Inclusion criteria are the following:

 Patients of both sexes aged 40 to 77 years.
 Predominant internal tibiofemoral knee osteoarthritis.
 Joint pain above 2.5 VAS points.
 Radiographic severity degrees 3 and 4 according to Ahlbäck scale.
 Values of body mass index between 20 and 33.
 Possibility for observation during the follow-up period.

Exclusion criteria are the following:

 Bilateral knee osteoarthritis which requires infiltration in both knees.
 Values of body mass index > 33.
 Polyarticular disease diagnosed.
 Severe mechanical deformity (diaphyseal varus of 4° and valgus of 16°).
 Arthroscopy in the last year prior to treatment.
 Intra-articular infiltration of hyaluronic acid in the past 6 months.
 Systemic autoimmune rheumatic disease (connective tissue diseases and systemic necrotizing vasculitis).
 Poorly controlled diabetes mellitus (glycosylated hemoglobin above 9%).
 Blood disorders (thrombopathy, thrombocytopenia, and anemia with Hb < 9).
 Undergoing immunosuppressive therapy and/or warfarin.
 Treatment with corticosteroids during the 6 months prior to inclusion in the study.

The enrolment finished on 29 October 2014 and the pilot study was completed on 10 June 2015.

In the first visit, an orthopedic surgeon conducted a clinical and radiographic assessment of each patient, including their medical history and a complete blood count. Moreover, the doctor delivered a booklet that contained detailed instructions and the knee injury and osteoarthritis outcome score (KOOS) questionnaire, which had to be completed by the patients at the baseline visit and before follow-up visits. Patients were allowed to consume acetaminophen, but it was restricted 48 hours before filling the questionnaires.

Patients were identified by a code number and scheduled to undergo the experimental procedure, which consisted of three treatments of PRP on a weekly basis. The first treatment included one PRP intra-articular infiltration and two PRP intraosseous infiltrations (femoral condyle and tibial plateau). The next two treatments were conventional intra-articular injections.

### 2.2. PRP Preparation

90 mL of venous blood was extracted from the patient in order to prepare the PRP and withdrawn into 9 mL tubes containing 3.8% (wt/V) sodium citrate. Blood was centrifuged at 580 g for 8 minutes at room temperature. The 2 mL plasma fraction located just above the sedimented red blood cells, but not including the buffy coat, was collected in a tube and carried to the injection room for use. This plasma fraction preparation contained a moderate concentration of platelets (2 to 3 times the concentration of platelets compared with peripheral blood, depending on the platelet count and size as well as the hematocrit) and an absence of erythrocytes and leukocytes [21]. To initiate the activation of platelets clotting, calcium chloride (10% wt/V) was added to the liquid PRP aliquots just before injection. All procedures were performed under sterile conditions.

### 2.3. Treatment

**Figure 1:**
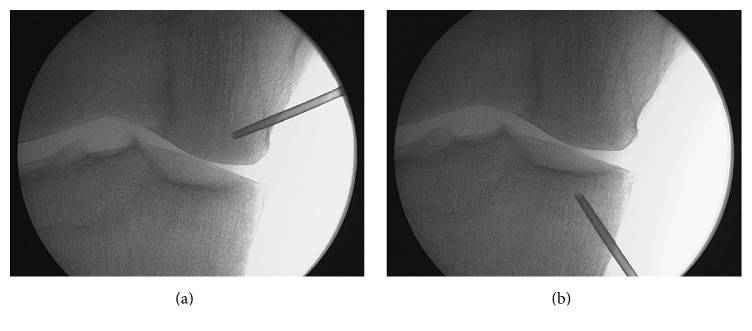
Fluoroscopic images. Intraosseous infiltration into the medial femoral condyle (a) and tibial plateau (b).

In the patient's first treatment, one PRP intra-articular injection and two PRP intraosseous injections were performed. Under anesthesiologist surveillance, sedation of the patient was induced by infusing a single dose of midazolam (0.03–0.05 mg/kg) and fentanyl (3.2 mg/kg), in a peripheral vein; single or repeated dose of propofol was also administered (1-2 mg/kg), depending on the duration of the infiltration. The degree of sedation was −4 or −5 on Richmond Sedation Scale. The patient was positioned in a supine position on an operating room table and two marks were drawn in the medial region of the knee, one located 2 cm proximal and the other located 2 cm distal to medial joint line; the infiltration area was prepared with a povidone-iodine solution. Local anesthesia was conducted by injecting 2 mL of 2% mepivacaine into the periosteum of condyle and tibial plateau. After evacuating the totality of the synovial fluid, 8 mL of PRP (the first intra-articular infiltration of a series of three) was infiltrated intra-articularly through the mid-point area of the femoropatellar region using a lateral approach in order to reach the joint space after lateralization of the patella. Intraosseous infiltrations were performed with a 13 G trocar used for bone biopsy, which was manually introduced into the bone and inserted 2 cm into the medial tibial plateau and medial femoral condyle. Once the trocars were placed in the desired position, 5 mL of PRP was infiltrated into subchondral bone of each structure. The control of trocar placements was facilitated by using a fluoroscope (Figure 1) [22]. After intraosseous infiltration is completed, ice is applied to the site. In the days after surgery, the patient can bear weight and take analgesics (acetaminophen) as required for pain. It is worth mentioning that the application of intra-articular and intraosseous infiltrations of PRP does not entail any reduction in physical activity and patients resume their daily activities few hours after the procedure is performed.

Two more intra-articular PRP infiltrations were performed 7 and 14 days after the first treatment. Moreover, the synovial fluid evacuated prior to the infiltrations was preserved for analysis.

### 2.4. Follow-Up

Patients were called for follow-up visits 2 and 6 months after the last treatment visit in order to conduct clinical evaluation. During these visits, the patient submitted the questionnaires given at baseline. A rheumatologist carried out a clinical examination and an evaluation of pain and function by visual analogue scale (VAS) and Lequesne Index, respectively. Acetaminophen consumption was also controlled.

### 2.5. Clinical Outcomes

The primary outcome was defined as the decrease in knee pain from the baseline to second month and sixth month (endpoint), according to the KOOS questionnaire. Furthermore, measurement of VAS and Lequesne Index was also evaluated; the secondary outcomes included the other areas of KOOS: symptoms, function in daily living (ADL), function in sport and recreation (sport/rec.), and knee related quality of life (QOL).

### 2.6. Safety Outcomes

To evaluate the safety of treatment, all complications and adverse events were assessed and reported during patient visits. Their nature, onset, duration, and severity were documented.

### 2.7. Biological Outcomes

Presence of mesenchymal stem cells (MSC) in synovial fluids before and one week after intraosseous infiltration was evaluated by flow cytometry and cultures of colony-forming cells (CFU-F). Concerning flow cytometry, each sample was immunophenotyped using an 8-color direct immunofluorescence technique. Concentrated cell suspensions were stained with the following combination of monoclonal antibodies (MoAb) in order to detect the expression of CD105/CD45/CD73/CD271/CD34/CD13/CD90/CD44: [Brilliant violet (BV) 421/orange chrome (OC) 500/fluorescein isothiocyanate (FITC)/phycoerythrin (PE)/peridinin chlorophyll protein-cyanin 5.5 (PerCP-Cy5.5)/PE-cyanin 7 (PECy7)/allophycocyanin (APC)/APCH7]. Regarding CFU-F assay, collected synovial fluids were diluted in phosphate buffered saline (PBS) and centrifuged in order to harvest the cellular content. The sample was used for colony-forming assay (CFU-F) and seeded on a 100 mm diameter culture plate. Seven days later, plating colonies were noted and counted by 0.5% crystal violet staining.

### 2.8. Sample Size Calculation

Power analysis was conducted to estimate the minimum sample size needed to achieve 80% power at a 5% level of significance for the primary outcome measures. An assumed effect size of 10 points (minimal clinically important change, MIC) with a standard deviation (SD) of 12 points was used [23]. This analysis suggested a minimum of 13 patients, expecting a dropout rate of 0.1.

### 2.9. Statistical Analysis

Demographic and medical variables (gender, age, and OA grade) were determined by the mean, standard deviation, range, and percent. For this study, a pair protocol analysis was used. Comparisons were performed by Student's *t*-test for paired-samples parametric data or Wilcoxon signed-rank test for paired-samples nonparametric data, after assessing the normal distribution of the samples by Shapiro-Wilk test. Data were considered statistically significant when *p* < 0.05. Statistical analysis was performed with SPSS 17.0 (SPSS, Chicago, IL).

## 3. Results

**Figure 2:**
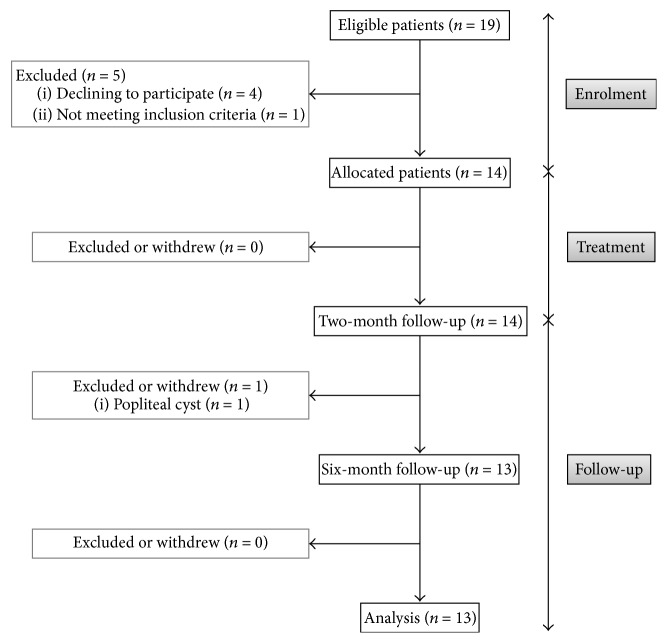
Enrolment and outcomes.

A total of 19 patients were considered eligible to participate in this study, and 14 patients were finally enrolled (Figure 2). Of the 5 excluded patients, four declined to participate and one presented predominant lateral osteoarthritis. Of the remaining 14 patients, 13 completed the study and one was excluded during the follow-up period due to a popliteal cyst.

Nine of the thirteen patients who finished the study were men and four were women, with a mean age of 62 ± 10 years (range: 47–75 years). Nine patients were diagnosed with OA III and five were diagnosed with OA IV, according to Ahlbäck scale (Table 1).

### 3.1. Clinical Outcomes

Table 1 summarizes results of primary and secondary outcome measures for the entire population that completed the study. Analysis of the primary outcome measure (as the decrease in knee pain from baseline to week 24, according to the KOOS questionnaire) showed a statistically significant improvement in pain reduction from 61.55 ± 14.11 at baseline to 74.60 ± 19.19 six months after treatment (*p* = 0.008). Eleven patients improved, and 8 patients reported minimal clinically important improvement (MCII) (Table 1). Depending on the osteoarthritis grade, eight of the 9 patients with degree 3 showed improvement as did 3 of the 4 patients with degree 4.

Regarding secondary outcomes, there was also a statistically significant improvement in all other areas of the KOOS (symptoms, *p* < 0.004; ADL, *p* < 0.02; sport/rec., *p* < 0.02; QOL, *p* < 0.02), as well as VAS score (*p* < 0.001) and Lequesne Index (*p* = 0.008).

**Figure 3:**
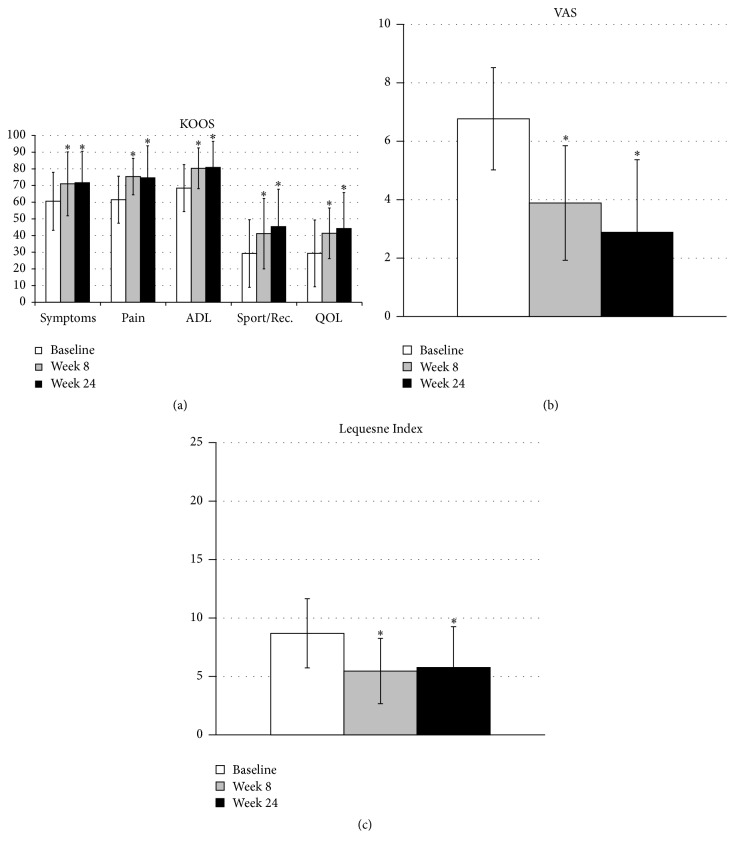
Clinical outcomes. KOOS (a), VAS (b), and Lequesne Index (c) at baseline, 8 weeks after treatment, and 24 months after treatment. ADL: function in daily living; sport/rec.: function in sport and recreation; QOL: quality of life. ^*∗*^ *p* < 0.05 with respect to basal level.

The improvement of the patients was observed at 8 weeks of follow-up, and it was maintained until week 24, when the study ended (Figure 3). The two patients who did not respond to treatment were indicated for a total knee arthroplasty.

Two patients reported 2 adverse events likely unrelated to the treatment. One of the patients experienced an episode of fever associated with flu episode, and the other reported exacerbation of knee pain three months after the treatment. Both events were mended satisfactorily by oral pharmacological treatment, which was allowed in the study. In addition, one patient was excluded because of a popliteal cyst caused by sports activity which was treated with fluid drainage and corticosteroid infiltration.

### 3.2. Biological Outcomes

Baseline levels of mesenchymal stem cells (MSCs) presented in synovial fluid were 7.98 ± 8.21 MSC/*μ*L, while one week after intraosseous infiltration the values significantly declined to 4.04 ± 5.36 MSC/*μ*L (*p* = 0.019) (Table 1).

Concerning cultures of colony-forming cells (CFU-F), a substantial reduction in the number of CFU-F was also observed one week after infiltration, namely, the number of CFU-F/mL before and after treatment of 601.75 ± 312.30 and 139.19 ± 123.61, respectively (*p* = 0.02) (Table 1).

## 4. Discussion

**Figure 4:**
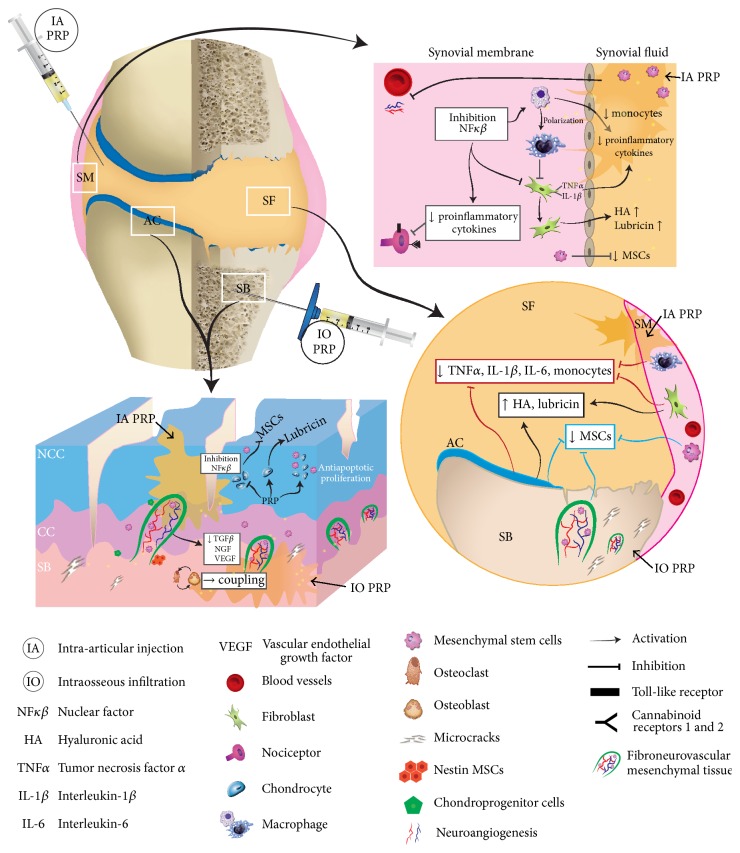
Mechanisms of intra-articular and intraosseous injections of platelet rich plasma. Depiction of a new strategy to treat severe knee OA by targeting different knee joint structures such as synovial membrane (SM), synovial fluid (SF), articular cartilage (AC) with noncalcified cartilage (NCC) and calcified cartilage (CC), and subchondral bone (SB) with intra-articular injections (IA) and intraosseous infiltrations (IO) of platelet rich plasma (PRP) [24]. This procedure reduces pain and mesenchymal stem cells (MSC) in SF, besides significantly improving knee joint function of patients with severe OA. We suggest that various growth factors, cytokines, and chemokines trapped in the fibrin network of PRP might inhibit the NF*κβ* on synovial macrophages, fibroblasts as well as on chondrocytes, thereby dampening the inflammatory response of SM and AC [15–18]. In addition, IO in subchondral bone, might buffer the excess of transforming growth factor *β*1 (TGF-*β*1) as well as restore hepatocyte growth factor (HGF) activity synthesized by osteoblasts, thereby leading to a new reestablished homeostatic balance between TGF-*β*1 and HGF [35–37]. The buffer effect of PRP on TGF-*β*1 signalling pathway in SB might reduce the presence of nestin MSCs in SF, likely associated with the shrinking of fibroneurovascular tissue in the SB, as an antifibrotic mechanism which has already been reported on other cell phenotypes [36, 37].

The combination of intra-articular and intraosseous injections of PRP is an* in situ* local biological “joint-centric” approach to treat severe KOA addressing the SM, SF, and superficial zone of AC by intra-articular injections of PRP and deep zones of AC and SB through PRP intraosseous infiltrations [24]. The significant pain reduction from baseline shown in these results is according to several studies which have shown the substantial pain reduction in patients with KOA treated with intra-articular infiltrations of PRP [20, 25–27]. However, some patients do not respond to this treatment, a result which converges with the severity of osteoarthritis [28–30]. These studies confirmed that patients with advance KOA such as Ahlbäck III type did not improve after intra-articular injections of PRP. Intra-articular drug delivery does not address the subchondral bone as a tissue target, which might be one of the reasons for this absence of response. In this study, we added intraosseous injections for the conventional intra-articular treatment to address the SB as one crucial tissue target in the treatment of severe KOA (Figure 4).

There are several potential mechanisms by which intra-articular injections and intraosseous infiltrations of PRP might reduce knee pain.* In vitro* and* in vivo* studies have reported that PRP and growth factors within it such as HGF, IGF-1, and PDGF suppress macrophage, fibroblast, and chondrocyte activation by inhibiting the NF*κ*B pathway, thereby dampening the synovial and articular cartilage inflammatory response [4, 15–17]. In addition, the significant amount of endogenous cannabinoids within PRP might act as ligands for cannabinoid receptors 1 (CB1) and 2 (CB2) of chondrocyte and synovium cells of OA patients, thereby supporting a pain and inflammation reduction by targeting the endogenous cannabinoid systems [2, 31–34]. On the other hand, the excessive presence of TGF*β*1 and VEGF in OA subchondral bone and articular cartilage could be a driving factor for changes in osteoblast-osteoclast coupling [7, 19, 35–37], which leads to a bone remodeling imbalance, NGF expression, and fibroneurovascular growth, all changes which might well contribute to pain [3, 7–9, 33, 35–37]. It is reasonable to speculate that the concurrent presence of, and a balanced ratio between, platelet-secreted TGF*β*1 and VEGF and plasma growth factors such as IGF-1 and HGF [37], all conveyed by PRP intraosseous infiltration, might buffer the excess of TGF*β*1 in SB as well as restoring HGF activity synthesized by osteoblasts. This new reestablished homeostatic balance between TGF*β*1 and HGF would reduce the synthesis of NGF, VEGF, and other inflammatory mediators, thereby contributing to the reduction of pain and hyperalgesia in severe stages of KOA [9, 36].

In this study, patients also showed a significant improvement in the secondary efficacy outcomes such as function in daily living (ADL), function in sport and recreation (sport/rec.), and knee related quality of life (QOL). This increased intolerable physical load might entail a positive chondroprotective and anti-inflammatory effect, since as several lines of evidence suggest, moderate mechanical loading of joints prevents cartilage degradation by suppressing the activation of NF*κ*B [38].

The significant reduction of MSC in SF after treatment with this novel PRP therapy is open to interpretation. Several studies have reported that the accumulation of MSCs in SF increases with the severity of osteoarthritis, joint damage, and the disease duration [39, 40]. Although the source of this MSC increase has not yet been determined, the most likely origin of the increased presence of MSC in SF of KOA patients might be the SM, the breakdown zone of superficial AC, and the SB [6, 7, 9, 39–41]. By adhering to SM, superficial AC, and SF and by gradually delivering various components such as IGF-1, HGF, PDGF, TGF-*β*1, and platelet microparticles (PM), intra-articularly injected PRP may influence macrophage M1 polarization towards M2 phenotype and modify the inflammatory status of chondrocytes and the superficial zone of AC by suppressing the NF*κ*B signaling pathway [15–17, 42]. By lowering the concentration of chemoattractant inflammatory cytokines in SF, PRP may well contribute to the inhibition of the MSC release and migration [4, 26, 43]. Another origin for SF MSCs might be the SB as a point of egress through the channels and vessels breaching the osteochondral junction, partially recruited by the osteoarthritic SF [7, 9, 43]. The buffer effect of PRP on TGF*β*1 signaling pathway in SB might reduce the presence of nestin MSCs likely associated with the shrinking of fibroneurovascular tissue of KOA subchondral bone as an antifibrotic mechanism which has already been reported on several cell phenotypes [36, 37]. Moreover, the process of cell* homing* whereby SF MSCs might be recruited to damaged areas of AC and take part in the* in vivo* repair of that cartilage might also contribute to MSCs reduction [44], just as the PRP fibrin network, containing fibronectin, IGF-1 and IGF-II, PDGF, SDF-1, and TGF*β*1 may exert a recruitment, homing, and chondrogenic-differentiation effect on subchondral mesenchymal progenitor cells [14, 45, 46].

This study has some limitations. First, a relatively small number of patients were enrolled in the study with no control group, all belonging to the same severe KOA phenotype stage. Second, the clinical follow-up of 6 months seems to be a short period to draw conclusive clinical indications. Third, an evaluation of patients with X-ray or MRI has been very useful to document eventual changes in the subchondral bone after PRP treatment. Finally, a mechanistic account of the significant pain and SF MSCs reduction experienced by the majority of patients is lacking. The first three limitations are inherent in the nature of the study.

## 5. Conclusions

In summary, targeting synovial membrane, synovial fluid, articular cartilage, and subchondral bone with intra-articular injections and intraosseous infiltrations of PRP reduces pain and MSCs in SF, besides significantly improving knee joint function in patients with severe knee OA, with no adverse event reported. This work aims to be a first step for further research in this field, both in basic research and in increasingly robust clinical trials.

## Figures and Tables

**Table 1:** Demographic data and biological and clinical outcomes.

Demographic data						
Patients	Total: *n*	Men: *n* (%)	Women: *n* (%)	Age: mean ± SD (range)	OA III: *n* (%)	OA IV: *n* (%)
	13	9 (69.23)	4 (30.77)	62.23 ± 9.6 (47–75)	9 (69.23)	4 (30.77)

Biological outcomes						
	Baseline: mean ± SD	One week after infiltration: mean ± SD	*p*			

MSC/*μ*L	7.98 ± 8.21	4.04 ± 5.36	0.019^*∗*^			
CFU-F/mL	601.75 ± 312.30	139.19 ± 123.61	0.012^*∗*^			

Clinical outcomes						
	Baseline: mean ± SD	Endpoint: mean ± SD	*p*	*δ*: mean ± SD (% change)	Improved patients: *n* (%)	Patients with MCII [22]: *n* (%)

KOOS pain	61.55 ± 14.11	74.60 ± 19.19	0.008^*∗*^	13.10 ± 14.89 (24.19 ± 40.07)	11 (84.62)	8 (61.53)
KOOS symptoms	60.56 ± 17.35	71.70 ± 18.82	0.004^*∗*^	11.14 ± 11.34 (19.73 ± 25.42)	11 (84.62)	8 (61.53)
KOOS ADL	68.44 ± 14.08	80.86 ± 15.58	0.022^*∗*^	12.45 ± 17.31 (23.25 ± 38.82)	11 (84.62)	8 (61.53)
KOOS sport/rec.	29.23 ± 20.29	45.38 ± 22.40	0.017^*∗*^	11.78 ± 11.54 (76.94 ± 115.23)	10 (76.92)	7 (53.84)
KOOS QOL	28.10 ± 19.75	39.28 ± 16.52	0.012^*∗*^	14.90 ± 22.03 (66.66 ± 72.64)	11 (84.62)	8 (61.53)
VAS	6.77 ± 1.75	2.88 ± 2.48	<0.001^*∗*^	−3.88 ± 2.82 (−55.04 ± 38.21)	11 (84.62)	10 (76.92)
Lequesne Index	8.69 ± 2.65	5.77 ± 3.49	0.008^*∗*^	−2.92 ± 3.35 (−31.18 ± 46.61)	10 (76.92)	

OA: osteoarthritis; MSC: mesenchymal stem cells; CFU-F: cultures of colony-forming cells; VAS: visual analogue scale; KOOS: knee injury and osteoarthritis outcome score; ADL: function in daily living; sport/rec.: function in sport and recreation; QOL: quality of life; *δ*: difference from baseline. MCII: minimal clinically important improvement; ^*∗*^ *p* < 0.05 with respect to basal level.
